# Bipolar Disorder Predisposition in a Greek Male With β-Thalassemia Trait: External Factors, Including COVID-19, and Other Clinical Implications

**DOI:** 10.7759/cureus.59303

**Published:** 2024-04-29

**Authors:** Sarah Tedesco, Katherine Reyes, Alec I Meiselman, Julian A Secondino, Nelya Tarnoverskyy

**Affiliations:** 1 Psychiatry, Richmond University Medical Center, Staten Island, USA; 2 Psychiatry, American University of Antigua, New York, USA

**Keywords:** hemoglobinopathy, genetic predisposition, covid, mediterranean, bipolar disorder with psychotic features

## Abstract

The present case study examines an adult male of Greek descent diagnosed with the β-thalassemia trait during adulthood. The individual had psychiatric symptoms after the sudden cessation of anabolic steroid injections, which had been utilized improperly for nearly a decade. Furthermore, the administration of an increased dosage of bupropion in conjunction with the absence of treatment for manic symptoms may have contributed to worsening his illness. The individual's contraction of COVID-19 and the subsequent discontinuation of steroid medication resulted in a notable psychosis despite the absence of any prior psychiatric conditions. Following initial therapy and hospitalization, which resulted in a stable discharge, the patient experienced a relapse due to later alterations in his medication. Consequently, this relapse necessitated a second admission to the hospital. The patient's therapeutic regimen consisted of a concurrent administration of lithium, antipsychotics, and an intense program of psychiatric counseling. This particular example highlights the distinctive connection between β-thalassemia and bipolar disorder, focusing on a Greek patient with the β-thalassemia trait and a genetic predisposition to mood disorders. The present study provides a comprehensive narrative of the patient's clinical progression, with particular emphasis on the impact of the β-thalassemia trait on his mental health trajectory. This observation highlights the limited availability of data about the interplay between hemoglobinopathies and mood disorders, hence emphasizing the need for further research in this niche intersection of genetics and psychiatry.

## Introduction

β-thalassemia trait, a prevalent hemoglobinopathy among those of Southeast Asian, Middle Eastern, and Mediterranean descent, has seen its carrier population grow in North America due to increased immigration [1]. This condition, characterized by a mutation in the β-globin gene, results in misshapen or deficient hemoglobin, thereby limiting red blood cells' oxygen-carrying capacity [2]. Altinoz and Ince posited that such alterations in hemoglobin's oxygen affinity might chronically influence neural tissue metabolism, potentially impacting psychiatric disease development [3]. Furthermore, Bocchetta identified an association between the β-globin gene, located on chromosome 11p15, and mental illnesses, including bipolar disorder, a psychiatric condition marked by significant fluctuations in a person's mood, energy, and ability to function that range from manic/hypomanic (abnormally happy or irritable mood) to depressive episodes (sad mood), with periods of neutral mood [4,5]. These episodes, which can last for several days, can disrupt daily life, leading to impaired judgment, reckless behavior, and strained relationships [6].

The interplay between COVID-19 and mental health disorders introduces an additional layer of complexity. The impact of COVID-19 on various organ systems, notably the central nervous system (CNS), where it influences angiotensin-converting enzyme 2 (ACE2)-expressing dopaminergic and serotonergic neurons [7], suggests a potential for psychiatric sequelae [8]. There are emerging case reports linking COVID-19 with the onset of mental disorders, yet the evidence, while biologically plausible, has yet to firmly establish a correlation. The hypothesis's novelty, its rarity, and the heterogeneity in psychosis assessments, along with reliance on anecdotal rather than empirical evidence, pose challenges to definitively linking COVID-19 and psychiatric conditions such as psychosis.

This case report of a 41-year-old Greek male, who exhibited late-onset psychosis following a COVID-19 infection and abrupt steroid cessation, highlights the potential cumulative impact of β-thalassemia trait and COVID-19 on mental health. His subsequent diagnosis with β-thalassemia trait during a later hospitalization raises critical questions about the interplay between hemoglobinopathy, infectious diseases, and psychiatric conditions. In presenting this case, we aim to contribute to the multidisciplinary understanding of how genetic disorders and infectious diseases intersect with mental health, especially within genetically predisposed populations. This report seeks to provide a comprehensive overview of the patient’s clinical presentation, psychiatric assessment findings, treatment interventions, and outcomes, with a focus on the potential roles that β-thalassemia and COVID-19 may play in the development and course of mental illness in predisposed patients of the Mediterranean population.

## Case presentation

The patient is a 41-year-old married male of Greek descent, who was brought to the comprehensive psychiatric emergency program (CPEP) following an episode of psychotic agitation, in the context of medication noncompliance. Upon initial assessment, the patient appeared calm and superficially cooperative with the evaluation. However, as the interview progressed, it became evident that he was guarded and evasive in his responses, providing vague and brief answers. Additionally, there was a noticeable escalation of agitation, throughout the duration of the interview, including psychomotor agitation as well as angry affect. He was also manifesting delusions of grandeur with a religious theme as he asserted statements such as “I am Jesus” and “God speaks with me.” Furthermore, he claimed that he had been trying to hurt "the devil" while at the gym and was the victim of a misunderstanding.

As per the patient's history, he lived with his wife and daughter at the time of his admission. The patient had graduated from high school, met all developmental milestones appropriately, and was presently unemployed. The patient had a history of multiple psychiatric hospitalizations. His first admission he had revealed a chronic history of anabolic steroid abuse, which he had discontinued abruptly, while simultaneously contracting COVID-19. Although he tested positive, he was asymptomatic, did not receive treatment for COVID-19 and tested negative for the virus by the end of his admission. While he had been discharged with depression after that admission, he shortly returned and was re-admitted with acute mania and psychosis, subsequently diagnosed with bipolar I disorder. The patient was admitted three times in one year with his fourth admission, currently being discussed, a year later. His admissions all reflect as being secondary to manic/psychotic and aggressive behavior (Table 1). Table 1 further provides details to his medications trials and changes.

**Table 1:** Hospitalizations With Medication Trials PO: by mouth; BID: twice daily, TID: three times daily, qAM: in the morning, qPM: in the evening, qHS: bedtime.

Hospitalization	Time Since Last Discharge	Reason for Admission	Medications Upon Admission	Medications Upon Discharge
1	----	Depressed mood after stopping steroids abruptly	Buspirone 10 mg PO BID	Bupropion 150 mg PO daily for depression tapered; Buspirone to 5 mg PO daily toward discontinuation
2	3 months	Acute mania and psychosis	Bupropion 150 mg PO daily; Buspirone 5 mg PO daily; Hydroxyzine 50 mg PO daily	Lithium Carbonate600 mg PO qAM and 900 mg PO qPM; Aripiprazole 20 mg PO for psychosis; Clonazepam 0.5 mg PO qPM for sleep
3	3 weeks	Aggression, mania, and psychosis	Quetiapine 50 mg PO qAM and 200 mg PO qPM; Lithium Carbonate 900 mg PO qPM; Benztropine 1 mg PO BID	Olanzapine 5 mg PO qAM and 10 mg PO qPM; Lithium Carbonate 900 mg PO qHS; Benztropine 1 mg PO BID
4	16 months	Aggression, mania, and psychosis	Lamotrigine 200 mg PO qHS; Lithium Carbonate 300 mg PO TID; Lurasidone 80 mg PO daily; Gabapentin 600 mg PO BID	Lithium Carbonate 600 mg PO qAM and 900 mg PO qPM for bipolar disorder; Haloperidol 5 mg PO qAM and 10 mg PO qPM for psychosis; Lamotrigine 150 mg PO qAM and 100 mg PO qPM for mood stabilization

As per the patient's wife, the patient had previously been prescribed Bupropion 150 mg by mouth (PO) daily for depression, Lithium Carbonate 900 mg PO twice a day for bipolar disorder, Lurasidone 80 mg PO daily for mood stabilization, and Gabapentin 600 mg PO twice a day as an adjunct for bipolar disorder. He reported minimal hours of sleep for at least over a week, averaging around seven to 10 hours a week. During that time, he also reported he was spending large amounts of money, had become very religiously preoccupied, and was non-compliant with his Bupropion for about three days. Due to the patient's presentation, in which he was acutely psychotic, endorsing auditory hallucinations at times, with religious and hypersexual preoccupations, delusions of grandeur, poor sleep, and tangential thought process, the decision was made to admit the patient to the hospital involuntarily for psychiatric stabilization.

**Figure 1:**
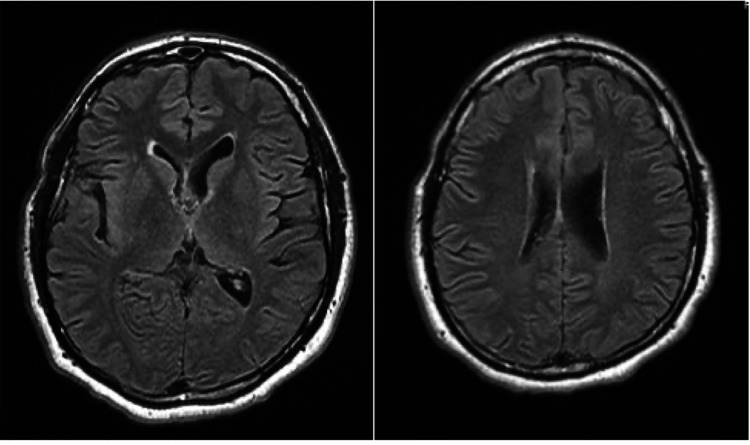
MRI Head Without Contrast No acute intracranial abnormality. Mild scattered T2 hyperintense foci in the bilateral frontal white matter.

A thorough medical workup was done, including a complete blood count which showed that the patient was slightly anemic and had low levels of free testosterone. Due to reports that his daughter suffered from immune thrombocytopenic purpura (ITP), as well as reports that he and his wife possibly had a hemoglobinopathy trait, and he was unable to clarify, the patient was then tested for the α and β-thalassemia trait. Ultimately, he tested positive for β-thalassemia minor. An MRI of the brain without contrast was performed that showed the presence of iron overloading (Figure 1). Apart from these results noted above, all other laboratory and imaging investigations were unremarkable. The patient was admitted under the diagnosis of decompensated bipolar I disorder with psychotic features. His vital signs included a heart rate of 104 beats per minute, blood pressure of 151/99 mmHg, respiratory rate of 18, and a temperature of 98.3F. The patient’s initial treatment regimen consisted of Lithium Carbonate 300 mg PO three times per day for mood stabilization, Lamotrigine 200 mg PO at bedtime, and Gabapentin 600 mg PO twice a day, and the patient's Lurasidone was increased from his home dose of 80 mg PO once a day to 80 mg PO in the morning and 40 mg PO in the evening.

On day two of the patient's admission, he began to share more with the team about his symptoms. The patient endorsed feelings of depression, increasing paranoia, religious preoccupation, and worsening frustration tolerance. The patient continued to state throughout this time that his reported insomnia was the result of his medication, Bupropion. The patient also admitted to missing a few doses of Bupropion, which led to him taking an extra dose of Bupropion before subsequently self-discontinuing all of his medication without consulting his psychiatrist. He displayed apathy and continued to minimize his symptoms. Additionally, the patient would attempt to rationalize his manic and aggressive behavior before admission.

By the third day of admission, the patient began endorsing chest pain in the middle of the night. Vital signs at that time were within normal limits except for a heart rate of 193 beats per minute, which was rechecked and found to be 212 beats per minute. The patient was hyperventilating, and the stat EKG showed a prolonged QTc interval of 487 ms. The medical team was consulted, and it was agreed to decrease Lurasidone from 120 mg per day to 100 mg per day, and the patient was started on Clonazepam 1 mg PO three times a day for impulsivity and anxiety with a plan to taper the patient off of Clonazepam prior to discharge.

On day four of admission, the patient continued to exhibit periods of grandiosity, elevated energy, and manipulative behavior with the goal to leave the hospital. It was around this time that the patient was started on Olanzapine 5 mg PO twice a day for continued psychosis and mania. By day six, the patient's Olanzapine was titrated up to 5 mg PO in the morning and 15 mg PO in the evening. The patient then began endorsing improved sleep and appetite, although he continued to display a flight of ideas, tangentiality, and poor insight into his illness.

On day eight of the patient's hospitalization, the decision was made to discontinue Olanzapine due to poor effect, and the patient was started on Haloperidol 5 mg PO three times a day for psychosis. Throughout the following days, it appeared Haloperidol significantly improved the patient's hostility, unpredictable behavior, and psychosis. The patient was less labile and grandiose and much easier to redirect. He would have bouts of hypersexuality toward his wife as well as sharing his clothing with others and religious preoccupations, but significantly less frequently with this medication. Between days 10 and 16, the patient was also able to recall events leading up to his initial manic break, including his impulsive spending on his daughter in fear of her hematologic conditions, “want to give her the best”, with catastrophic thoughts, eventually weaned to more linear and mindful cognition when discussing these events through the course of admission.

By his 16th day of admission, the patient, although still religiously preoccupied, no longer felt like he was “the Messiah” or “Jesus Christ” and no longer believed to possess magical healing powers. Despite retaining some religious preoccupation, he displayed a future-oriented mindset, expressing a desire to take his family on a holiday vacation. His behavior, insight, judgment, and impulse control continued to improve steadily. The patient was discharged on a medication regimen that included Lithium Carbonate 600 mg PO in the morning with an evening dose of 900 mg PO for bipolar disorder. His lithium level before discharge resulted at 0.82 mEq/L. He was also discharged on Haloperidol 5 mg PO in the morning and 10 mg PO in the evening for psychosis. Lamotrigine was titrated to 150 mg PO in the morning and an evening dose of 100 mg PO for mood stabilization. The patient's discharge diagnosis was bipolar disorder with psychotic features.

The patient transitioned to an outpatient treatment program where he would initially receive weekly therapy sessions for the first month followed by biweekly sessions thereafter. Since discharge, he has consistently attended his outpatient appointments. The patient no longer exhibits psychotic features and has an appropriate mood, thought process, and thought content. His insight, judgment, and impulse control also appears to have improved. He required some changes in his medication regimen. However, Haloperidol, Lithium Carbonate, and Lamotrigine remain a part of his daily regimen.

## Discussion

The genetic linkage between mental disorders and β-thalassemia, exemplified by the case of a 41-year-old Greek male with bipolar disorder onset following a COVID-19 infection who also carries the β-thalassemia trait, underscores the intricate interplay of genetic, infectious, and environmental factors in psychiatric illness. Altinoz and Ince proposed a notable imprinting mechanism, where the β-globin gene, located in proximity to key neurological genes such as those for dopamine receptor D4, brain-derived neurotrophic factor (BDNF), and tyrosine hydroxylase, could underlie the co-occurrence of bipolar disorder and β-thalassemia [3]. This hypothesis suggests that linked genes on the same chromosome, implicated in both conditions, may be inherited together, highlighting a potential predisposition to mental health disorders among individuals with hemoglobinopathies, particularly in Mediterranean populations where β-thalassemia is prevalent. This case, featuring a late-onset diagnosis of bipolar disorder alongside an acute psychotic episode in a patient with the β-thalassemia trait, reinforces the need for further investigation into the risk of psychiatric illness in patients with β-thalassemia. Additionally, it emphasizes the importance of considering genetic screening for β-thalassemia in individuals predisposed to psychiatric conditions, a move that could represent a significant advancement in psychiatry as our understanding of this genetic linkage expands.

The significantly higher prevalence of the β-thalassemia trait among bipolar patients, as reported by Bocchetta, underscores a notable genetic vulnerability within bipolar subgroups [4]. This study revealed a significant decrease in mean corpuscular volume (MCV) of hemoglobin (<80 μ^3^) among bipolar patients (25.0%) compared to non-bipolar groups (18.1%), with statistical significance (p = 0.02; relative risk = 1.38). Such a decrease in MCV, indicative of microcytosis, is clinically significant as it suggests impaired hemoglobin's oxygen-carrying capacity, potentially affecting brain oxygenation and contributing to psychiatric symptomatology. Furthermore, a significant portion of bipolar patients (16.4%) exhibited the heterozygous β-thalassemia trait, a rate substantially higher than in the non-bipolar subgroup (9.9%, p = 0.01; relative risk = 1.65). This finding highlights an increased risk of bipolar disorder within Mediterranean populations carrying the β-thalassemia trait, suggesting that hemoglobinopathies may serve as potential contributory factors to psychiatric conditions.

The intersection of microcytosis and bipolar disorder prompts further consideration of how hemoglobinopathies may contribute to the development and worsening of psychiatric conditions. It also accentuates the complex interplay of genetic, environmental, and infectious elements, such as COVID-19 and steroid abuse, in the pathogenesis and aggravation of psychiatric disorders. The overlap of anemia-induced symptoms, such as sleeplessness and mood disturbances, with those of bipolar disorder, suggests that the β-thalassemia trait, especially in the context observed in the patient's case, might facilitate the onset and intensification of late-stage bipolar disorder and psychosis. This hypothesis is supported by the understanding that impaired oxygen transport due to β-thalassemia could exacerbate neurophysiological vulnerabilities, leading to psychiatric manifestations [9].

Given the treatability of anemia and its potential role in exacerbating bipolar disorder symptoms in patients with β-thalassemia, exploring the biological connections between these conditions becomes imperative. This exploration could involve clinical trials investigating the impact of anemia management on mood stabilization and psychiatric outcomes in this patient population. Such research could provide valuable insights into mitigating psychiatric sequelae in susceptible individuals, potentially offering new therapeutic targets that address both the hemoglobinopathy and its psychiatric manifestations.

By delving into the mechanisms through which genetic predispositions like the β-thalassemia trait interact with environmental and infectious triggers to influence psychiatric health, future research can pave the way for comprehensive, multifaceted treatment approaches. These approaches could not only improve management strategies for bipolar disorder in patients with β-thalassemia but also enhance our understanding of the broader implications of genetic and environmental interplay in psychiatric disease.

The intersection of COVID-19 infection with psychiatric conditions, particularly new-onset psychosis, has emerged as a significant area of concern within the medical community. A systematic review conducted by Moccia et al. illuminates this intersection, revealing an association between COVID-19 and the emergence of psychosis in patients who previously had no psychiatric history [8]. This finding is critical, suggesting the potential role of COVID-19-induced inflammatory responses and direct neurological effects in precipitating psychiatric symptoms. The phenomenon underscores an urgent need for further investigation into the pathophysiological mechanisms underpinning COVID-19's impact on mental health, advocating for a deeper understanding that could guide both clinical management and therapeutic interventions.

Compounding the complexity of COVID-19's impact on psychiatric health is the consideration of steroid treatments, which, while pivotal in managing severe manifestations of the virus, may inadvertently contribute to psychiatric symptomatology. The administration of steroid injections, a common recourse in the treatment of COVID-19, stands out as a double-edged sword, potentially exacerbating or even triggering psychiatric conditions. This aspect of treatment underscores the intricate challenge of balancing the benefits of steroid use against the risk of adverse psychiatric effects, highlighting the necessity for vigilant monitoring and personalized care strategies in treating COVID-19 patients with a predisposition to psychiatric disorders.

Further complicating this landscape are detailed case studies that shed light on the diverse manifestations of COVID-19-related psychosis. Narratives, such as those presented by Moccia et al. and Al-Busaidi, offer a window into the clinical complexity of such cases, ranging from somatic delusions to severe psychosis in patients devoid of previous psychiatric conditions [8,10] . These cases not only illustrate the clinical challenges posed by COVID-19-related psychiatric symptoms but also emphasize the paramount importance of personalized and multifaceted treatment approaches. These narratives serve as compelling evidence for the necessity of a tailored approach in psychiatric care, especially for patients experiencing COVID-19-related psychiatric episodes.

At the heart of these discussions is the recognition of the multifactorial nature of psychiatric manifestations in patients with COVID-19, where factors such as genetic predispositions (e.g., the β-thalassemia trait), the viral infection itself, and treatment modalities (e.g., steroids) intertwine. The case at hand exemplifies this complexity, highlighting how these interrelated factors can create a conducive environment for the exacerbation of psychiatric conditions. This recognition demands an integrated approach to patient care, one that holistically addresses the gamut of contributing factors to offer the most effective treatment and support.

The potential interplay of genetic predispositions, infectious diseases, and environmental factors, as observed in the context of COVID-19, underscores the urgent need for comprehensive, personalized psychiatric care. Specifically, the β-thalassemia trait highlights a critical area of concern, raising questions about heightened susceptibility to environmental insults, such as COVID-19. This genetic condition, with its profound hematological implications, may predispose individuals to a more severe response to infections, underscoring a dual challenge of managing systemic inflammation and immune reactions alongside underlying vulnerabilities. Such a scenario likely exacerbates psychiatric symptomatology, drawing a direct line between genetic predispositions and increased vulnerability to infectious diseases.

Building on the understanding of heightened susceptibility, it is crucial to delve deeper into how the confluence of β-thalassemia and COVID-19 directly impacts mental health, particularly concerning the development of mood disorders. The cumulative physiological stress exerted by β-thalassemia, coupled with the psychological burdens introduced by COVID-19 infection, creates a potent environment conducive to the emergence of mood disorders. This case exemplifies the intricate manner in which a genetic hematological disorder intertwined with an acute infectious disease not only disrupts physical health but also profoundly impacts mental well-being. The synergistic effect of β-thalassemia and COVID-19, acting in concert, may significantly elevate the risk and intensity of mood disorders. This observation underscores the necessity of adopting a holistic treatment paradigm that meticulously addresses both the physical and psychiatric sequelae stemming from these conditions.

This complex interplay between genetic factors, infectious diseases, and their psychiatric outcomes necessitates targeted research to unravel the underlying mechanisms. Understanding these mechanisms is pivotal in crafting interventions that mitigate psychiatric sequelae in susceptible populations, emphasizing the indispensable role of tailored psychiatric care. Such care should integrate genetic insights, infectious disease management, and environmental considerations, highlighting the multifaceted nature of psychiatric conditions in the context of COVID-19. The imperative for a holistic approach becomes even more pronounced, advocating for a multidisciplinary strategy in treating and understanding psychiatric illnesses in patients with unique vulnerabilities like those presented by β-thalassemia in the era of COVID-19. As the medical community continues to navigate the multifaceted relationships between these factors, it becomes increasingly clear that an integrated approach to research and clinical practice is essential for advancing our understanding and management of psychiatric illnesses in the era of COVID-19.

## Conclusions

This case highlights the importance of considering demographics, medical history, and genetic factors in treating mood disorders and psychosis, especially in Mediterranean populations. The patient's unique presentation, including a first manic episode, COVID-19 diagnosis, and β-thalassemia trait, emphasizes the interaction between genetic vulnerabilities and environmental factors. A link between Mediterranean populations with hemoglobinopathies like β-thalassemia and mood disorders suggests a need for nuanced psychiatric care. Further research is needed to explore the role of genetic hematological disorders in mental illnesses and develop personalized interventions. Overall, this case stresses the necessity of holistic psychiatric approaches tailored to individual backgrounds and medical histories.
